# Influence of Residential Environments on Older Adults’ Social and Physical Activities: A Scoping Review

**DOI:** 10.1093/geroni/igaf122.3005

**Published:** 2025-12-31

**Authors:** Seokyung Park, Wenjin Wang, Xuemei Zhu

**Affiliations:** Texas A&M University, College Station, Texas, United States; Texas A&M University, College Station, Texas, United States; Texas A&M University, College Station, Texas, United States

## Abstract

This scoping review examines how older adults’ residential environments—homes of community-dwelling older adults and long-term care facilities—impact their social and physical activities, which are key components of active aging. While prior research on active aging has explored broad environmental influences, such as nature, pollution, neighborhood environments, and social environments, relatively few studies have focused on older adults’ homes and long-term care communities. A systematic search of four databases (PubMed, PsycINFO, CINAHL, and Web of Science) was conducted, using 14 keywords related to older adults, residential environments, social activity, and physical activity. A total of 3,333 studies were screened following the Preferred Reporting Items for Systematic Reviews and Meta-Analyses (PRISMA) guidelines, with 70 meeting the eligibility criteria for data extraction. Older adults’ social and physical activities have been studied across various types of housing, ranging from large-scale multifamily housing to single-family homes. The findings were categorized into four spatial scopes within residential environments: (1) indoor spaces, (2) indoor-outdoor transitional areas, (3) outdoor spaces within the property or site, and (4) immediate surroundings (e.g., sidewalks connected to buildings and nearby houses). This scoping review clarifies the current state of research on how residential environments correlate with older adults’ social and physical activity at a more granular spatial scale, highlighting existing knowledge gaps. The findings underscore the need for further investigation to better inform and refine the design of age-friendly residential environments that support older adults’ well-being and active lifestyles.

